# An Untraceable Data Sharing Scheme in Wireless Sensor Networks

**DOI:** 10.3390/s19010114

**Published:** 2018-12-31

**Authors:** Dong Chen, Wei Lu, Weiwei Xing, Na Wang

**Affiliations:** 1 School of Software Engineering, Beijing Jiaotong University, Beijing 100044, China; luwei@bjtu.edu.cn (W.L.); wwxing@bjtu.edu.cn (W.X.); 2 School of Computer Science, Beijing University of Posts and Telecommunications, Beijing 100876, China; nawang@bupt.edu.cn

**Keywords:** untraceable, wireless sensor networks, security, data sharing

## Abstract

With the wide application of wireless sensor networks (WSNs), secure data sharing in networks is becoming a hot research topic and attracting more and more attention. A huge challenge is securely transmitting the data from the source node to the sink node. Except for eavesdropping the information stored in the packages, the adversary may also attempt to analyze the contextual information of the network to locate the source node. In this paper, we proposed a secure data sharing approach to defend against the adversary. Specifically, we first design a secret key mechanism to guarantee the security of package delivery between a pair of nodes. Then, a light-weighted secret sharing scheme is designed to map the original message to a set of shares. Finally, the shares are delivered to the sink node independently based on a proper random routing algorithm. Simulation results illustrate that our approach can defend against the eavesdropping and tracing-back attack in an energy-efficient manner.

## 1. Introduction

Wireless sensor networks (WSNs) consist of quite a number of tiny, green and smart nodes. In general, these nodes can automatically compose a connected network, though each node can directly communicate with only several neighbors which can greatly save the energy of the nodes. Based on the properties of economy and flexibility, WSNs have been extensively employed in military surveillance, industrial control, disaster management, forest fire detection and traffic monitoring [1,2,3]. In general, the nodes that find the target are defined as the source nodes and they are responsible for reporting the target-related information to the sink node. A huge challenge is how to securely deliver the information in a relay manner without leaking the location privacy of the monitored target.

In real life, the target-related information is hidden in packages before being transmitted. It is likely that the adversary can obtain the location, status and some other description information of the target if he can decrypt the packages. Many schemes have been proposed to protect the security of data transmission between the nodes in the network [4,5]. Therefore, it is extremely difficult for the adversary to decrypt the transmitted packages. Except for content privacy, the adversary may also attempt to collect the contexture privacy of the network and find the target by analyzing the network traffic. In general, there is always a hot-spot around the source node and the adversary can find the source node by performing hot-spot attack [4].

Recently, many schemes have been designed in the literature to protect the source-location privacy in distributed sensor networks. Part of these schemes are designed based on the global adversary model [6,7], i.e., the adversary can monitor the whole network and knows all the radio actions. However, this is unrealistic for a large network considering economic factors. Moreover, if the adversary can indeed monitor the whole network thoroughly, we can infer that he can directly monitor the target in this area and need not locate the target by analyzing the network traffic.

A more practical adversary model is the local adversary model [4,5,8,9,10,11]. In this model, the adversary can monitor a local area with a similar size to that of a sensor node’s communication area and knows all the radio actions in this area. In this model, the adversary finds the source node by tracing back hop-by-hop. Roughly speaking, the adversary starts from the sink node and if he finds that node *A* sends a message, he moves to node *A* and continues to monitor the surrounding environment. If the adversary finds that node *B* sends a message, he moves to node *B*. By iterating the above process, the adversary can finally find the source node. The detailed trace back attack in this paper will be given in Section 4. To defend against the local adversary, routing-based source-location privacy protection schemes [9,10,11] are proposed. In these schemes, the source node does not directly send the message to the sink node. The source node first sends the message to a random agent node and then the message is transferred by the agent node to the sink node. In this way, the routing paths are diversified, and it is difficult for the adversary to receive a continuous flow of the packages to find the source node. However, if the sensing range of the adversary is large or the target stays in a location for a long time, it is highly possible that the adversary can find the target [8].

To further improve the security of the source node, in this paper, we mainly focus on a novel attack named hot-spot attack [4] and propose an untraceable data sharing scheme in WSNs. In the proposed scheme, we designed a secret sharing scheme based on the congruence equations and it can map the original message to a set of shares that are much shorter in length. Based on the shares, a cloud is constructed around the source node in which all the sensor nodes are indistinguishable in statistics. Theoretical analysis and simulation show that our cloud is much more energy-efficient than that of existing schemes. On the border of the cloud, the shares are independently transmitted to the sink node by a proper random routing algorithm. Finally, the sink node recovers the original message from the received shares and then the data delivery process from the source node to the sink node is completed. Simulation results illustrate that our scheme can strongly protect the location of the source node in an energy efficient way.

The rest of this paper is organized as follows: In Section 2, we summarize the related studies and mainly focus on the local-adversary-based source location protection schemes. The network model is discussed in Section 3 and Section 4 introduces the smart adversary and trace back attack in detail. In Section 5, we present the untraceable data sharing scheme and evaluate its performance in Section 6. Finally, Section 7 concludes this paper.

## 2. Related Studies

Yasmin et al. [12] proposed an efficient and secure framework for authenticated broadcast/multicast by sensor nodes as well as for outside user authentication. Moreover, many secure data transmission schemes [13,14,15] were designed for cluster-based networks. To solve the orphan node problem, Lu et al. [13] proposed a secure and efficient data transmission scheme based on identity-related digital signatures. Though these encryption-based data delivery schemes can properly hide the information in each package, they cannot prevent the adversary from locating the monitored targets by analyzing the transmission paths of the packages. Based on secure data transmission techniques, many source-location privacy protection schemes have been proposed.

To defend against the global adversary, each source node in the network and all the nodes on the routing paths of the packages need to periodically send the real packages. Meanwhile, the other nodes need to send dummy packages in the same rhythm. Considering that all the nodes in the network have the same radio pattern, the adversary cannot accurately locate the source node but can monitor all the radio actions of all the nodes in the whole network. It is a tradeoff between the time delay of package delivery and the amount of data transmission. Shao et al. [6] decrease the time delay by making the sensor nodes send the real package as soon as possible while keeping them indistinguishable from the dummy packages. In this way, though the time delay greatly decreases, the data transmission amount does not increase. To decrease the number of dummy packages, each cluster head can filter the dummy packages in [16] and, similarly, the scheme in [17] selects a set of sensor nodes as proxy nodes to filter the dummy packages.

Except for global adversary, some other schemes were designed for local adversary. In phantom routing algorithm [8], the message generated by the source node first randomly walks for *k* steps before being transmitted to the sink node to defend against the local adversary. Considering that the fake source nodes are variable with a random location, it is difficult for the adversary to locate the real source node. In some cases, the walk steps conceal with each other and the message is close to the source node. To make each step effective, the authors provide two variants namely sector-based directed random walk and hop-based directed random walk. In this way, the packages can be far from the source node and this increases the security of the source node. The performance of some routing algorithms in defending against jamming attack can be found in [18]. Recently, Mahmoud et al. [4] proposed a cloud-based source-location-privacy protection scheme. Before sending the package to the sink node, the source node first constructs a cloud with irregular shape and all the nodes send the packages with the similar pattern to conceal the source node. Simulation results illustrate that these schemes can protect the source node to some extent and, meanwhile, the data transmission amount increases. However, all the above schemes provide an opportunity for the adversary to sketch the outline of the suspicious region and this is a huge threat to the security of the source node.

## 3. Network Model

**Figure 1:**
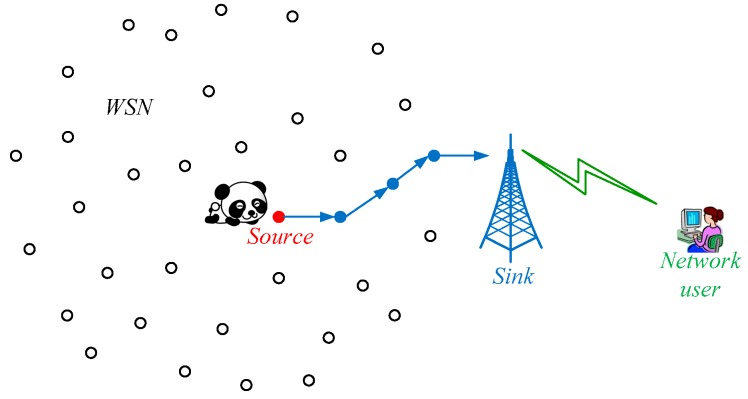
Network model.

As shown in Figure 1, the considered network is composed of a sink node and a set of homogenous sensor nodes, which are randomly scattered in the interested area. In this paper, we assume that all the sensor nodes in the network are static and strictly resource-limited in terms of power, communication, storage, and computation. However, they are capable of sensing, processing data, receiving and sending data, and hence they can fulfill sophisticated tasks in a collaborative manner which is discussed in Section 4. After deployment of the network, these sensor nodes intelligently form a connected network based on hello mechanism, and each pair of nodes can bi-directionally communicate with each other if their distance is not larger than *R_c_*. If a pair of nodes can directly communicate with each other, they are defined as two neighboring nodes. To keep the transmitted data confidential from the adversary, we further assume that each node negotiates secret keys with all the neighbor nodes and the sink node. Then, all the delivered packages in the network are encrypted based on the keys before being transmitted.

As the transmission radius of the sensor nodes is very small, the sensor nodes cannot directly send data to the network operator. Fortunately, they can communicate with the network user through the sink node that is much powerful. We also assume that the sink node is stationary and, however, it has sufficient storage and computation capabilities to store and process the collected data of the network. In addition, the sink node has enough power to communicate with the network user through the Internet, 3G/4G or satellite communication techniques. Note that the sink node is the sole destination of all the data generated by the sensor nodes and the data packages are delivered to the sink node in one hop or multi-hops. To protect the monitored data, they are encrypted before being packed into data packages.

In this paper, we employ the deployed network to monitor wild animals such as pandas. Specifically, the network needs to accurately record basic vital signs of the pandas, such as location, body temperature, heart rate, sleep quality, and even blood pressure in the near future. The basic descriptions of the pandas can be properly fused further to estimate the physical status of the pandas and hence we can take good care of them. To collect these data flexibly, each panda needs to wear a smart tiny wearable device that can communicate with the neighboring sensor nodes. This is reasonable considering that the size of the wearable device is constrained and hence sending the data directly to the sink node is impractical. For the sake of convenience, we assume that the communication range of the wearable device is also *R_c_*.

Though the wearable device may be able to communicate with a set of sensor nodes in a specific location, it sends the data only to the nearest sensor node in order to save energy and prolong the lifetime of the network. Meanwhile, the nearest sensor node becomes the source node. The source node is responsible for delivering the data to the sink node in a relay manner. The nodes keep salient if they are not the source node or locate on the routing paths of the packages. Apparently, the source node is naturally near to the pandas and once the adversary finds the source node, he can locate the pandas easily. This is a great threat to the safety of the pandas and we need to protect the location privacy of the source node.

## 4. Smart Adversary Model and Trace-Back Attack

The adversary plan to hunt the pandas by analyzing the network traffic. To achieve this goal, the adversary employs a set of monitoring devices and we denote them as hunter nodes. Each node consists of an antenna, spectrum analyzer, storage device, processor and mobile device to perform the trace-back attack. As the hunter nodes are well equipped, we assume that they have enough resources to store, process, transmit the monitoring data of the network traffic. A hunter node can intercept the packages transmitted in the monitored area and accurately locate a sender’s location by measuring the angle of arrival as well as the strength of the signal. However, we assume that the adversary cannot decrypt the packages to capture the plaintexts. The sensing range of the hunter node is denoted as *R_S_*. As the adversary does not need to accurately transfer the signals into digital codes, we can infer that *R_S_* is some larger than *R_c_*. To reflect the relation between *R_S_* and *R_c_*, we introduce a parameter ρ(ρ≥1) into our scheme and set $R_{s}=\rho R_{c}$. Note that, though the hunter nodes can locate the senders of the data, they cannot determine the receiver of the data considering that any node in the transmission area of the sender can be the potential receiver.

In the initial phase of trace-back attack, the hunter nodes are deployed around the sink node and monitor the surrounding signals. Once a package crosses the monitored area of a hunter node, in theory, the hunter can find that, at most, nearly 2ρ sensor nodes orderly send the package. Then, the hunter node needs to predict the direction of the former nodes that delivered the package and moves forward in the predicted direction by a proper distance *d*. In this paper, we assume that $d=R_{s}$.

**Figure 2:**
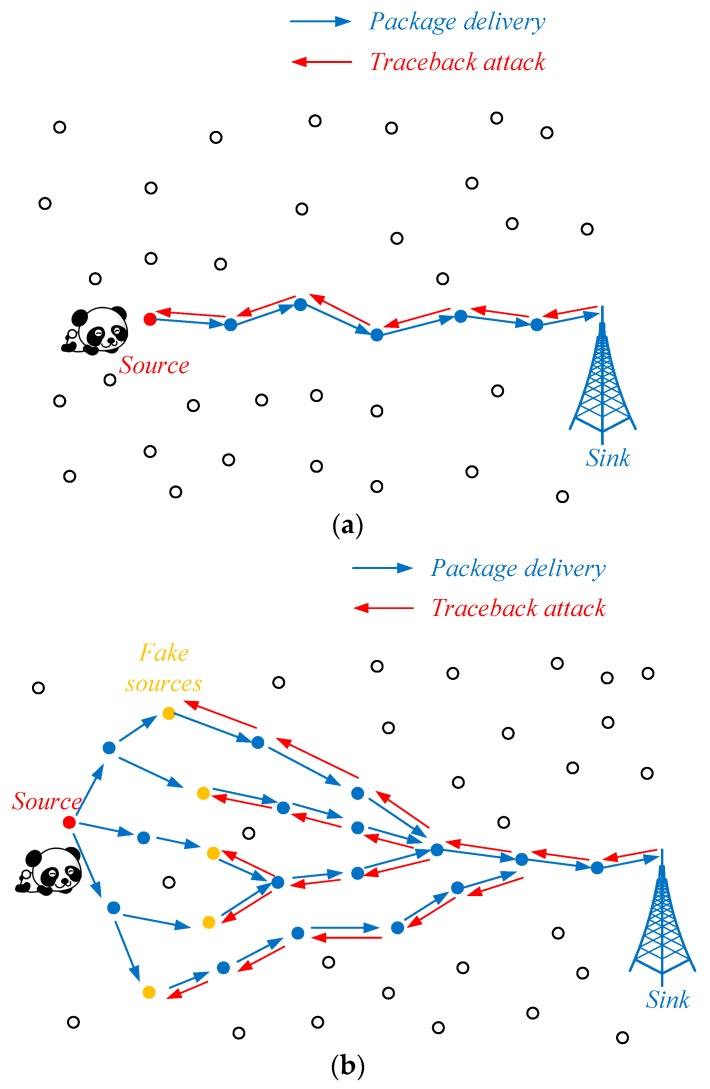
The trace-back attack in different networks. (**a**) Networks without source-location privacy protection; (**b**) networks with source-location privacy protection.

In practice, the pandas spend more time in some areas and less time in other areas. For example, they may tend to live in an area due to the availability of food, water, shadow, shelter, etc. In this case, the hunter nodes can easily find the source node in a collaborative manner if the network employs ordinary routing algorithms, such as the shortest path routing algorithm, Greedy Perimeter Stateless Routing (GPSR) [19], directed diffusion [20], etc. In the worst case, the adversary can even find the pandas by employing only one hunter node as shown in Figure 2a. If the network employs some proper source-location privacy protection schemes such as phantom routing algorithm [8] or anonymous cloud structure [4], the hunter nodes cannot directly locate the source node. However, the adversary can easily sketch the suspicious region that covers some pandas as shown in Figure 2b. In the process of tracing back, the range of the predicted direction increases with the increasing of the distance between the sink node and the hunter node. Then, more hunter nodes are needed to monitor all the possible areas that the packages may cross. In other words, the adversary needs to put more hunters on the routing path as the monitored area moves far from the sink node. Finally, the hunters can always find the border of the suspicious region. Under the assumption of the phantom routing algorithm, the hunters can form a circle around the panda. Specifically, the center of the circle is near to the location of the panda and its radius is about *k* times of the average distance between a pair of sensor nodes. In the cloud-based scheme, the hunters can locate the cloud.

Once the adversary successfully locates a suspicious region, a good strategy is scattering all the hunter nodes in this region to monitor the signals in fine-grained manner. In the limit case, we can treat the adversary in this region as a global adversary and assume that he knows all the radio actions of the sensor nodes in the suspicious area. Under this assumption, most existing schemes cannot defend against the smart adversary and the pandas are exposed to the adversary.

## 5. Secure Data Sharing between the Source Node and Sink Node

**Figure 3:**
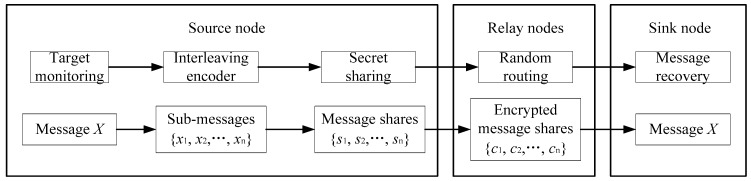
Process of data sharing between the source node and sink node.

To securely transmit the data to the sink node and defend against the smart adversary, we propose an untraceable data sharing scheme in wireless sensor networks. The flowchart of our scheme is presented in Figure 3. Initially, the source node monitors the target and generates message X that contains all the necessary information about the pandas. Then, we divide message X into a set of sub-messages $x_{1},\;x_{2},\cdots ,x_{n}$ and map them to a set of message shares $s_{1},\;s_{2},\cdots ,s_{n}$. An anonymous cloud is constructed based on the approach proposed in [4]. At the border of the cloud, the fake source nodes are responsible for sending these shares to the sink node independently. In the package delivery process, we employ a random routing algorithm to diverse the routing paths. Finally, the sink node recovers message X based on the received shares and, the data sharing process between the source node and the sink node is completed. The detailed process of delivering a package from the source node to the sink node is given in the following.

### 5.1. Message Splitting

**Figure 4:**
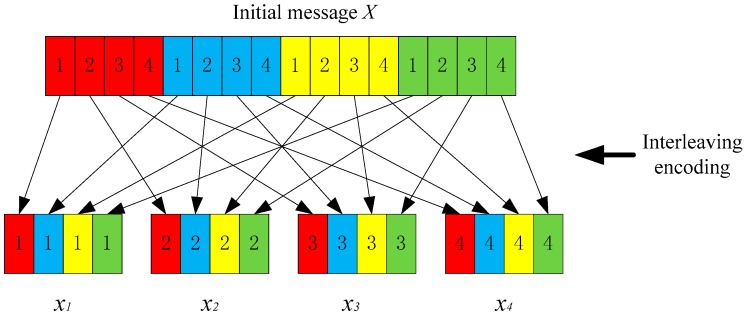
Message splitting based on interleaving encoder.

Initially, the source node generates message X and then we map X to a set of message segments through interleaving encoder. In this paper, we attempt to design a (t,n)-secret sharing scheme and hence the original message X needs to be split to exactly t sub-messages with equal lengths. For convenience sake, we assume that X is denoted as a binary number and the size, |X|, where X is defined as the number of bits. If the size of X cannot be divided by t, we fill X by a set of specific symbols at the tail until it can be divided into t equal-length sub-messages. Then, we split X to a set of sub-messages through an interleaving encoder. As an example, in Figure 4, we split X to four sub-messages, $x_{1},x_{2},x_{3},x_{4}$, and each sub-message contains a part of message X. Moreover, each sub-message is composed of four message fragments which are discontinuously extracted from X. In this way, each sub-message is of no clear semantic information. In other words, even if the plaintext of a sub-message is captured by the adversaries, they cannot get valuable information about message X. However, the sink node can recover X by an interleaving decoder once it can get $x_{1},x_{2},x_{3},x_{4}$.

### 5.2. Light-Weight Secret Sharing Scheme

Having got the sub-messages, $x_{1},x_{2},\cdots ,x_{t}$, of X by an interleaving encoder, we construct the shares of X based on Equation (1) as follows:

(1) $$s_{i}=\left\{ \begin{array}{ll} s_{1}+\cdots +s_{i-1}+ix_{i}+x_{i+1}+\cdots +x_{t}\mathrm{mod}p, & if\;1\leq i\leq t\\ s_{1}+2^{i-t-1}s_{2}+\cdots +t^{i-t-1}s_{t}\mathrm{mod}p, & if\;t<i\leq n \end{array}\right..$$

In Equation (1), p is defined as the largest binary number with size |X|/t, i.e., $p=2^{\left|X\right|/t}$. Then, we can infer that the size of $s_{i}$ is always not larger than |X|/t which is much smaller than that of |X|. This greatly increases the energy efficiency of constructing the cloud around the source node and we will theoretically analyze it in Section 6.1. Now, we prove the correctness and security of our scheme in Theorems 1 and 2, respectively.

**Theorem** **1.** *Any* T(T≥t) *shares of* X *can recover message X.*

**Proof.**  We prove Theorem 1 by considering two cases.

Case 1: We first prove that the first t shares, $s_{1},s_{2},\cdots ,s_{t}$, constructed by Equation (1) can recover message X. In this case, the shares are calculated as follows:

(2) $$\left\{ \begin{array}{l} s_{1}=x_{1}+x_{2}+\cdots +x_{t}\mathrm{mod}p\\ s_{2}=s_{1}+2x_{2}+\cdots +x_{t}\mathrm{mod}p\\ \;\;\vdots \\ s_{t}=s_{1}+s_{2}+\cdots +tx_{t}\mathrm{mod}p \end{array}\right..$$

We can present Equation (2) in matrix manner as shown in Equation (3):

(3) $$\left(\begin{array}{c} s_{1}\\ \vdots \\ s_{i}\\ \vdots \\ s_{t} \end{array}\right)=\left(\begin{array}{ccccc} a_{11} & \cdots & a_{1j} & \cdots & a_{1t}\\ \vdots & & \vdots & & \vdots \\ a_{i1} & \cdots & a_{ij} & \cdots & a_{it}\\ \vdots & & \vdots & & \vdots \\ a_{t1} & \cdots & a_{tj} & \cdots & a_{tt} \end{array}\right)\left(\begin{array}{c} x_{1}\\ \vdots \\ x_{i}\\ \vdots \\ x_{t} \end{array}\right)=M\left(\begin{array}{c} x_{1}\\ \vdots \\ x_{i}\\ \vdots \\ x_{t} \end{array}\right).$$

Based on Equation (2), we can calculate all the coefficients in matrix M as shown in the follows:

(4) $$a_{ij}=\left\{ \begin{array}{l} \begin{array}{cc} 2^{i-1}+2^{i-j-1}(j-2), & 2\leq j<i \end{array}\\ \begin{array}{cc} 2^{j-1}+j-1, & j=i \end{array}\\ \begin{array}{cc} 2^{i-1}, & i<j\leq t \end{array}\\ \begin{array}{cc} 2^{i-2}, & j=1,i>1 \end{array} \end{array}\right..$$

To prove that $s_{1},s_{2},\cdots ,s_{t}$ can recover X, we need to prove that matrix M is invertible and it theoretically equals to the fact that the determinant of M, i.e., |M|, is a non-zero number. We execute the following Algorithm 1 on matrix M and we can always get a diagonal matrix. As a consequence, we can prove that the determinant of matrix M equals to t! (i.e., |M|=t!) which is not zero. In other words, we can uniquely get $x_{1},x_{2},\cdots ,x_{t}$ based on Equation (2) once we get $s_{1},s_{2},\cdots ,s_{t}$.
**Algorithm 1:** MatrixTransformationInput: Square matrix MOutput: Lower triangular determinant1.Count the size of M which is composed of t rows and columns
2.For i=2 to t3.$M\left(i,:\right)=M\left(i,:\right)-2^{i-1}*M\left(1,:\right)$;4.End5.For j=2 to t6.M(:,j)=−M(:,1)+M(:,j);7.For k=2 to j−18.M(k,:)=M(k,:)−M(k,j)/M(j,j)∗M(j,:);9.End10.For l=j+1 to t11.$M\left(l,:\right)=M\left(l,:\right)-2^{l-j-1}*M\left(j,:\right)$;12.End13.End

Case 2: Then, we prove that any t shares can recover message X. Without loss of generality, we denote the shares as $s_{k_{1}},s_{k_{2}},\cdots ,s_{k_{i}},s_{t+k_{i+1}},s_{t+k_{i+2}},\;\cdots ,s_{t+k_{t}}$. The first i(1≤i<t) shares are selected from $s_{1},s_{2},\cdots ,s_{t}$ and the other t−i shares are selected from $s_{t+1},s_{t+2},\cdots ,s_{n}$. Based on Equation (1), we can calculate $s_{t+1},s_{t+2},\cdots ,s_{n}$ as follows.

(5) $$\left\{ \begin{array}{l} s_{t+1}=s_{1}+s_{2}+\cdots +s_{t}\mathrm{mod}p\\ s_{t+2}=s_{1}+2s_{2}+\cdots +ts_{t}\mathrm{mod}p\\ \;\;\vdots \\ s_{n}=s_{1}+2^{n-t-1}s_{2}+\cdots +t^{n-t-1}s_{t}\mathrm{mod}p \end{array}\right..$$

Under the above assumption, we can obtain the following equation. To prove that $s_{k_{1}},s_{k_{2}},\cdots ,s_{k_{i}},s_{t+k_{i+1}},s_{t+k_{i+2}},\;\cdots ,s_{t+k_{t}}$ is equal to $s_{1},s_{2},\cdots ,s_{t}$, we need to prove that matrix D is an invertible matrix.

(6) $$\left(\begin{array}{c} s_{k_{1}}\\ \vdots \\ s_{k_{i}}\\ s_{t+k_{i+1}}\\ \vdots \\ s_{t+k_{t}} \end{array}\right)=\left(\begin{array}{ccccccc} 0 & \cdots & 1 & \cdots & 0 & \cdots & 0\\ \vdots & & \vdots & & \vdots & & \vdots \\ 0 & \cdots & 0 & \cdots & 1 & \cdots & 0\\ 1 & \cdots & k_{1}^{k_{i+1}-1} & \cdots & k_{i}^{k_{i+1}-1} & \cdots & t^{k_{i+1}-1}\\ \vdots & & \vdots & & \vdots & & \vdots \\ 1 & \cdots & k_{1}^{k_{t}-1} & \cdots & k_{i}^{k_{t}-1} & \cdots & t^{k_{t}-1} \end{array}\right)\left(\begin{array}{c} s_{1}\\ \vdots \\ s_{i}\\ s_{i+1}\\ \vdots \\ s_{t} \end{array}\right)=D\left(\begin{array}{c} s_{1}\\ \vdots \\ s_{i}\\ s_{i+1}\\ \vdots \\ s_{t} \end{array}\right).$$

We can compute determinant of D and find that

(7) $$|D|=\underset{u>v,u,v\neq k_{1},\cdots ,k_{i}}{\Pi }(u-v)\underset{v\neq k_{1},\cdots ,k_{i}}{\Pi }v^{k_{i+1}-1}\neq 0.$$

Therefore, the matrix D is invertible and $s_{1},\;s_{2},\ldots ,\;s_{t}$ can be linearly expressed by $s_{t+k_{i+1}},s_{t+k_{i+2}},\cdots ,s_{t+k_{t}}$. Based on Case 1, we prove that the equations in Case 2 also have a unique solution. By combining Case 1 and Case 2, Theorem 1 is proved.  □

Based on Theorem 1, we can guarantee that the sink node can recover the original message X once at least t shares are received. Now, we analyze the security of the secret sharing scheme and prove its security in Theorem 2. In the network, the adversary may eavesdrop and decrypt the shares to recover X. However, we can prove that even the adversary successfully decrypts a set of the shares, they cannot recover X.

**Theorem** **2.** *Any* T(T<t) *shares of* X *cannot recover message X.*

**Proof.**  Assume that the adversary successfully gets the plaintexts, $s_{1}^{\prime },\;s_{2}^{\prime },\cdots s_{T}^{\prime }$, of T(T<t) shares. Based on Equation (1), the adversary can construct a set of equations with t variables $x_{1},x_{2},\cdots ,x_{t}$ as shown in Equation (8).

(8) $$A=H_{T\times t}B,$$

where $A={\left(s_{1}^{\prime },\;s_{2}^{\prime },\cdots s_{T}^{\prime }\right)}^{\prime }$, $B={\left(x_{1},x_{2},\cdots ,x_{t}\right)}^{\prime }$. Let F be a field and $H_{T\times t}$ be a matrix over F. We further assume that all the numbers in A and B are over filed F. Consider the augmented matrix, $HA_{T\times \left(t+1\right)}$, of $H_{T\times t}$ and we can in infer that $HA_{T\times \left(t+1\right)}=\left(H_{T\times t}\;A\right)$. Based on the ranks of $H_{T\times t}$ and A, we prove Theorem 2 by the following two cases:

Case 1: The rank of $H_{T\times t}$ is smaller than that of $HA_{T\times \left(t+1\right)}$. In this case, the equation set has no solution and the adversary cannot recover message X. This may happen when the adversary gets the wrong numbers of $s_{1}^{\prime },\;s_{2}^{\prime },\cdots s_{T}^{\prime }$.

Case 2: The rank of $H_{T\times t}$ equals the rank of $HA_{T\times \left(t+1\right)}$. In this case, there are T equations and t variables. Therefore, the adversary cannot accurately recover message X. We consider the worst case and assume that T=t−1, i.e., the adversary gets t−1 shares. In this case, the adversary can get |F| lawful solutions.

By combining Case 1 and Case 2, we can infer that it is impractical for the adversary to recover message X by T(T<t) shares for a field of a large size. Theorem 2 is proved.  □

In our scheme, the shares are delivered to the sink node independently and it is extremely difficult for the adversary to capture more than t shares at the same time. Consequently, our secret sharing scheme is safe.

### 5.3. Cloud Construction Based on Message Shares

**Figure 5:**
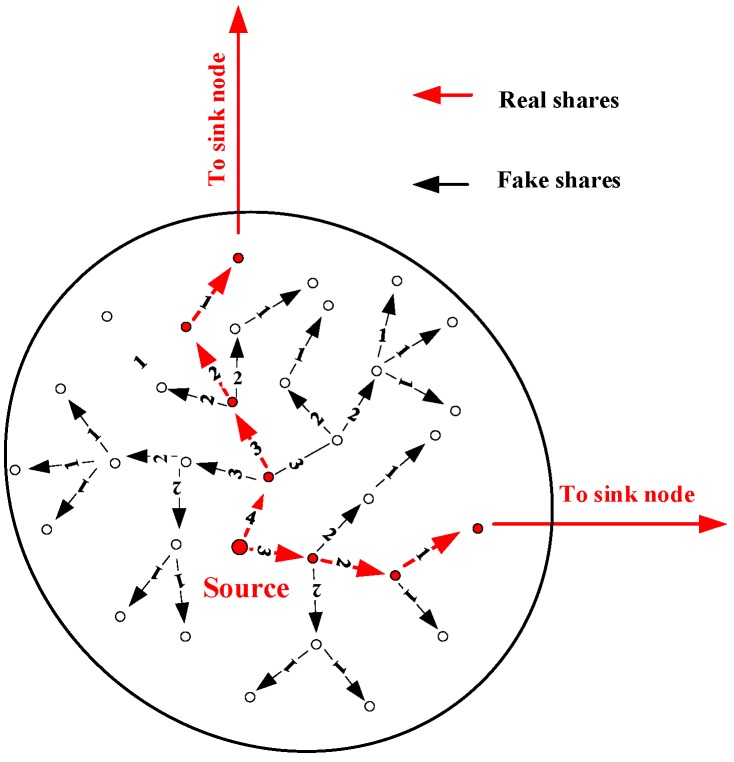
An example of cloud with two real shares.

Different from existing schemes, we employed the message shares rather than the original message to construct an anonymous cloud with irregular shape. For a message X, a set of message shares are derived, and together they recover X. To decrease time delay of message X, the best strategy is transmitting the shares to the sink node at the same time. In our scheme, the source node first assigns a random number k to each share and the share can walk exactly for k steps. Then, the source node sends the shares of a message to the neighbors randomly at the same time. If a node receives a real share, it first updates k by k−1 and hence the share can be further transferred for k−1 steps. Meanwhile, the node generates a set of fake shares with the same length with the real share. Moreover, these fake shares are randomly delivered to the neighbors of the node and they can also walk in the cloud for k−1 steps. If a node receives a fake share, the node first checks the number of steps and if the share cannot further walk in the cloud, it just drops the share. Otherwise, the node subtracts the step number of the share by one and then sends the share to its neighbors. As shown in Figure 5, an example of the cloud with two real shares is provided and the shares can randomly walk for 4 and 3 steps, respectively.

To make the nodes in the cloud statistically indistinguishable, all the nodes in the cloud employ the scheme in [6] to send the real shares or fake shares with a proper time delay. Specifically, the time delay is generated by a random distribution. However, to decrease time delay, the node transfers the real shares as soon as possible without breaking the statistical rule. In this way, all the nodes in the cloud have similar radio behaviors and hence the adversary cannot locate the real source node easily.

### 5.4. Data Delivery from the Source Node to the Sink Node

**Figure 6:**
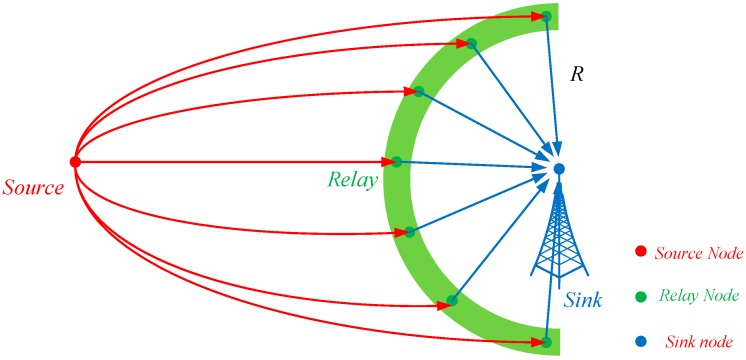
The possible area of the relay nodes.

At the border of the cloud, the fake source nodes send the shares to the sink node independently. To hinder the adversary locating the cloud easily, we integrate the All-direction Random Routing algorithm (ARR) [21] into our scheme. In the ARR scheme, the relay node is defined as the nearest node to the virtual location that is randomly selected by the source node. To balance the security and energy-efficiency of our scheme, the fake source node employs only one relay node and the possible area of the virtual location is presented in Figure 6. Initially, the real source node randomly selects a parameter R for message X and adds the parameter to all the shares of X. Then, the fake source node selects the virtual location based on parameter R. Specifically, the virtual location can be any point on the half circle with the source node as the center and R as the radius.

In theory, the adversary needs to first find the relay node, the fake source node and, finally, the real source node. As shown in Figure 6, the shares of message X are transmitted to the sink node from different directions. Therefore, it is almost impossible for the local adversary to locate the relay nodes, let alone the fake source node and the real source node. For different real source nodes and messages, the fake source nodes, relay nodes are totally different. The adversary cannot receive continuous package flows and hence the adversary can hardly trace back to the cloud. However, the adversary may occasionally locate the cloud sometime and, in this case, he still cannot find the real source node, because all the nodes in the cloud are indistinguishable.

Both simulation and theoretical analysis show that our network has a similar performance with typical WSNs in terms of energy consumption about the surrounding nodes of the sink node. Specifically, the nodes near to the sink node in both our network and a typical network have a similar life time. This can be explained by the fact that though more packets are delivered to the sink node, they are much shorter in length compared with the original messages. Overall, the total transmission amount of data that is received by the sink node is similar and considering that the locations of the targets are uniformly randomly generated in the network, the nodes around the sink node have a similar workload with that in typical networks.

## 6. Performance Evaluation

In this section, we thoroughly evaluate the performance of the proposed scheme by both theoretical analysis and simulation. In Section 6.1, we theoretically analyze the energy efficiency of our scheme, especially the data transmission amount of the cloud. In Section 6.2, we evaluate the performance of the proposed scheme in terms of security and energy efficiency based on ns-3 discrete event simulator. In the simulation, we set (t,n) as (4,7) if there is no specific declaration.

### 6.1. Theoretical Analysis

In existing cloud-based source-location privacy protection schemes, the cloud is constructed based on the original message X. In this paper, we assumed that the description data, X, of a panda is l bits and the head of a package is always $l^{\prime }$ bits, i.e., the total length of the package is $l^{\prime }+l$ bits. In existing cloud-based schemes, all the nodes periodically send packages that have the same of $l^{\prime }+l$ bits and the dummy messages are employed to hide message X. In a cloud with m nodes, the cloud needs to totally transmit and receive $m*\left(l^{\prime }+l\right)$ bits data in a period. If the packages walk k steps in average, $m*\left(l^{\prime }+l\right)*k$ bits data are transmitted because of message X. In our scheme, we design a (t,n)-secret sharing scheme and employ the shares to construct the cloud. It can be calculated that the length of the shares is l/t bits. The head of a package contains the basic geographic information about the destination and is necessary for all the packages. Therefore, the total length of the packages in our cloud is $l^{\prime }+l/t$. In the same cloud with existing schemes, the cloud needs to totally transmit and receive $m*\left(l^{\prime }+l/t\right)$ bits data in a period. Similarly, $m*\left(l^{\prime }+l/t\right)*k$ bits data are transmitted because of message X in our scheme.

Except for constructing the cloud, some extra energy is consumed in the process of delivering the packages from the source node to the sink node. We assumed that the distance between the fake source node and the sink node is $k^{\prime }$ hops. In the existing scheme, the data transmission amount is $(l^{\prime }+l)*\;k^{\prime }$ bits. In our scheme, the data transmission amount can be calculated as $\left(l^{\prime }+l/t\right)*n*k^{\prime }$.

**Figure 7:**
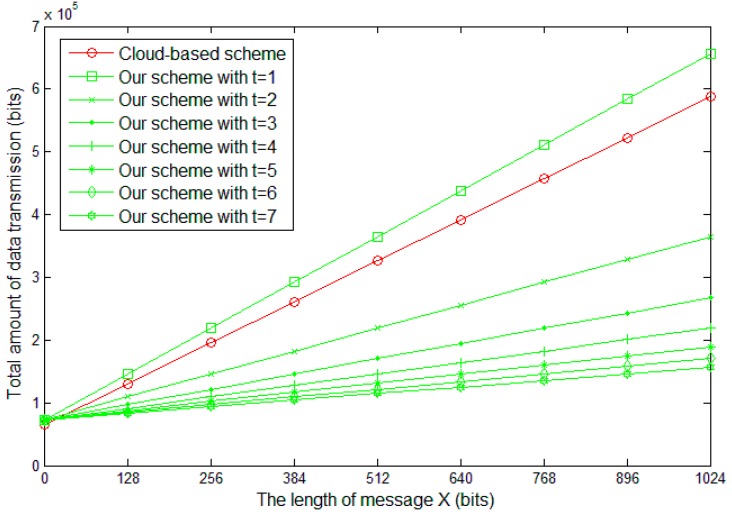
Total data transmission amount with different parameters.

By combining the above two phases, the total data transmission amount is $m*\left(l^{\prime }+l\right)*k+(l^{\prime }+l)*\;k^{\prime }$ in the existing cloud-based scheme. In our scheme, the total data transmission amount is $m*\left(l^{\prime }+l/t\right)*k+\left(l^{\prime }+l/t\right)*n*k^{\prime }$. For convenience sake, we set $m=100,\;l^{\prime }=128,\;k=5,\;k^{\prime }=10,\;n=7$ and the simulation results are presented in Figure 7.

It can be observed that with the increasing of l, the data transmission amount of both cloud-based scheme and our scheme increases. This is consistent with the analysis result. In our scheme, various records of the panda are hidden in message X and, without the specific statement, the length of X is assumed as 1024 bits in the following.

Except for total data transmission amount, the balance of nodes’ workload also affects the performances of these schemes. As discussed in Section 5.4, the nodes around the sink node in our network have a similar data transmission workload with that of nodes in typical WSNs. For each network, an interesting observation is that the nodes near to the sink node consume more energy than the nodes far from the sink node. This is reasonable considering that all the data need to be delivered to the sink node and the hence the nodes near to the sink node need to deliver more data. A proper solution to balance the workload of all the nodes in the network is that we can scatter slightly more nodes around the sink node.

### 6.2. Simulation

We compared our scheme with the shortest path routing algorithm, phantom routing algorithm and the cloud-based scheme in terms of privacy protection and energy-efficiency. In the simulation, 4000 sensor nodes were randomly scattered in a 3500 m × 3500 m square region. The communication range, $R_{c}$, of the sensor nodes was set as 50 m and we set the parameter ρ=2. The sink node was located in the center of the area. Initially, the location of the panda was randomly selected in the network and it slowly moved in the network with a speed 1 m/s. The wearable devices collected the information of the panda every 1 s and then the data were transmitted to the sink node. Moreover, we assumed that the packages in the cloud can be transmitted for 8 steps.

**Figure 8:**
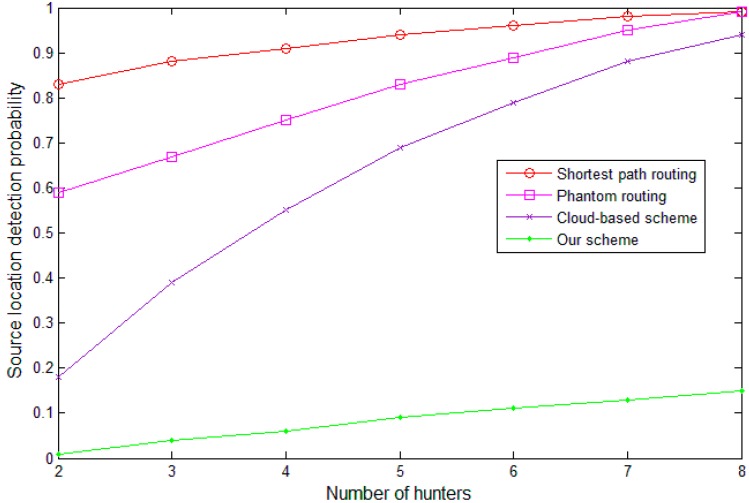
The probability of successfully locating the source node.

We employed the probability that the adversary can locate the source node in the simulation period to estimate our scheme. The probability was calculated by the number of times that the adversary can locate the source node to the total time of runs. As shown in Figure 8, with the increasing of the hunters’ number, the source-location detection probability increases for all the four schemes. The shortest path routing algorithm cannot protect the source location properly. Phantom routing algorithm performs better because of the random walk phase. However, the adversary can easily locate the source node by employing several hunters together. Though the cloud-based scheme can defend against the adversary if the number of hunters is small, it cannot defend the smart adversary model if the number of hunters is large. Our scheme performs the best because it is extremely hard for the adversary to find the cloud let alone the source node.

**Figure 9:**
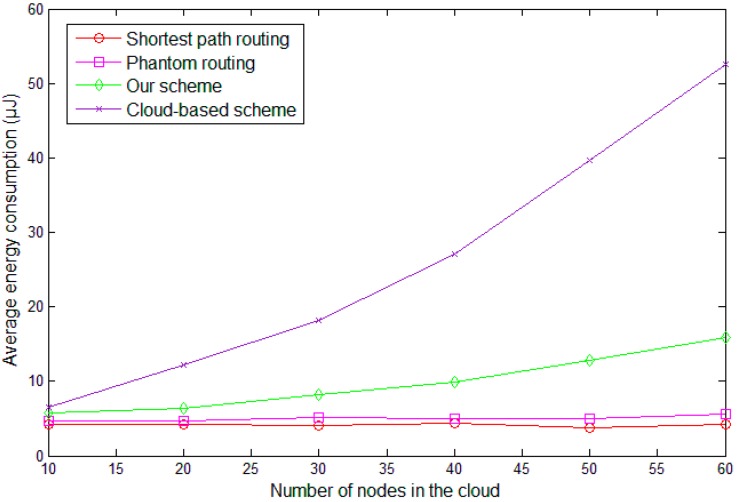
Energy consumption with different sizes of the cloud.

In Section 6.1, we theoretically analyzed the data transmission amount of our scheme. Though most energy of the network was consumed in delivering the data, some extra energy was consumed in processing these data. The total energy consumption of the network is presented in Figure 9. It can be observed that the average energy consumption of our scheme is slightly larger than that of the shortest path routing algorithm and phantom algorithm, and it is much more energy-efficient compared with the cloud-based scheme.

**Figure 10:**
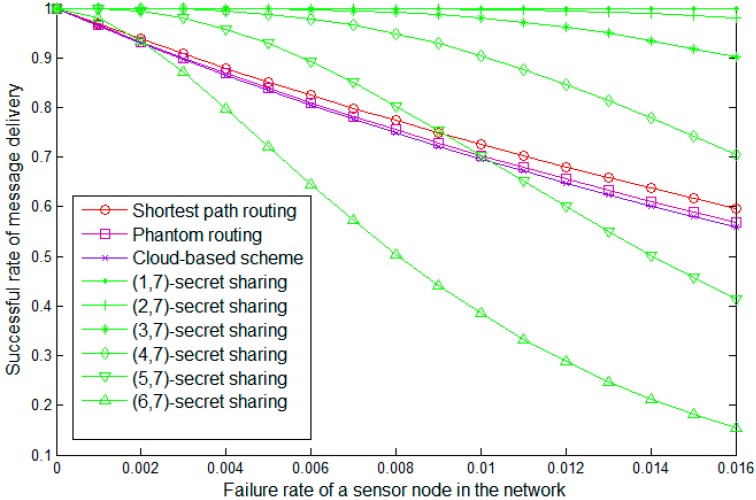
Successful rate of message delivery between the source node and the sink node.

Our scheme can protect the source-location-privacy in an energy-efficient manner and this is the main effect of our scheme. An interesting side-effect of our scheme is that the success rate of message delivery increases. This can be explained by the fact that we integrated the secret sharing scheme into our scheme. The simulation result is presented in Figure 10.

It can be observed that with the expending of the network’s size, the success rates of all the schemes decrease. In a larger network, the packages need to be relayed by more steps and hence the rate of package drop increases. The shortest path routing algorithm has the best performance because the packages can be delivered to the sink node with the least hops. The phantom routing algorithm also has a similar performance. Considering some extra walk steps in the random walk phase, the successful rate of the phantom routing algorithm is lower than that of the shortest routing algorithm. The cloud-based scheme has the worst performance. This can be explained by the fact that the packages walk in the cloud for a long distance and hence the hops greatly increase. Though the packages in our scheme also walk in the cloud, the secret sharing scheme improves the performance of our scheme. The sink node can recover the original message based on a set of shares rather than all the shares and this improves the tolerance of node failure.

## 7. Conclusions

In this paper, we designed a secure data sharing scheme in WSNs which can be used to protect source location privacy. Our scheme is novel, and it is designed by integrating a secret sharing scheme and the random routing technique. Specifically, a light-weight secret sharing scheme is proposed based on congruence equations. The original message generated by the source node is mapped to a set of shares and the shares are transmitted to the sink node independently. Considering that the routing paths are of a great variety even for a constant source node, it is almost impossible for the adversary to trace back to the source node. The security of the information delivered between a pair of nodes is guaranteed by an authentication mechanism. Simulation results show that our scheme can strongly protect the privacy of the source node and the confidentiality of the information transmitted in the network. In addition, the employment of the secret sharing scheme greatly improves the tolerance of node failure in the process of delivering packages from the source node to the sink node.
